# PVA-assisted metal transfer for vertical WSe_2_ photodiode with asymmetric van der Waals contacts

**DOI:** 10.1515/nanoph-2023-0398

**Published:** 2023-08-28

**Authors:** Xiaohui Song, Zhen Liu, Zinan Ma, Yanjie Hu, Xiaojing Lv, Xueping Li, Yong Yan, Yurong Jiang, Congxin Xia

**Affiliations:** Henan Key Laboratory of Photovoltaic Materials, School of Physics, Henan Normal University, Xinxiang 453007, China; Department of Electronic and Electrical Engineering, Henan Normal University, Xinxiang 453007, China

**Keywords:** WSe_2_, graphene, metal transfer, van der Waals contact, photodiode

## Abstract

The vertical electronic and optoelectronic devices based on 2D materials have shown great advantages over lateral devices, such as higher current density, faster switch speed, and superior short-channel control. However, it is difficult to fabricate vertical device with conventional metal deposition methods due to the aggressive process usually results in damage to the contact region. Here, we develop a simple and effective metal transfer technique and fabricate p-type and n-type WSe_2_ transistors by using metals with different work functions and subsequently create a vertical WSe_2_ transistors with a 18-nm-thick channel, which retain good gate coupling effect. Furthermore, a vertical WSe_2_ photodiode is constructed with graphene and Pt as asymmetric van der Waals (vdW) contacts. The work-function difference between graphene and Pt generates a built-in electric filed, leading to a high current rectification over 10^5^. Under 405 nm laser illumination, the device exhibits excellent self-powered photodetection properties, including a high responsivity of 0.28 A W^−1^, fast response speed of 24 μs, and large light on/off ratio exceeding 10^5^ at zero bias, which surpass most of the vdW photodiodes. This work demonstrates that the metal transfer technique is a promising strategy for the construction of high-performance vertical optoelectronic devices.

## 1 Introduction

Recently, vertical electronic and optoelectronic devices based on 2D materials have received considerable attention, in which the source electrode, semiconductor channel material, and drain electrode are vertically stacked together [1–6], and the transport direction of charge carriers is parallel to the direction of electric field generated by the gate electrode. In this case, the carriers are collected vertically rather than laterally, and hence the channel length is determined by the thickness of the semiconductor, which can be easily down-scaled to sub-10 nm without involving sophisticated lithography process. Therefore, the vertical configuration is particularly suitable for the fabrication of ultra-short-channel FETs. Compared with the laterally contacted 2D devices, the vertical geometry can offer great advantages, such as higher current density, faster switch speed, and superior short-channel control under a low operating voltage [4, 7]. In addition, in view of the unique charge transport direction, the vertical geometry also affords exceptional mechanical flexibility [2, 3, 8]. Diverse vertical devices have been reported based on graphene and other 2D semiconductors, including light-emitting diodes [9, 10], Schottky diodes [11, 12], tunneling transistors [1, 13], [14], [15], and photovoltaic cells [8, 16, 17], demonstrating great potential of vertical design in the development of future electronic and optoelectronic devices.

For the vertical geometry devices, due to the source and drain electrode fully cover the whole channel, the role of metal contacts becomes very important, and the properties of metal–semiconductor interfaces can even dominate the device performance, including maximum current, on/off ratio, carrier mobility, polarity, and so on [18–24]. Therefore, high-quality metal–semiconductor contact is crucial to the performance of vertical geometry devices. However, the atomically thin structure of 2D materials leads to their crystal lattice to be easily damaged during metal deposition processes, such as electron-beam (or thermal) evaporation and sputtering, which produce considerable defects and surface states at the metal–semiconductor interface, leading to strong Fermi-level pinning effect and/or the formation of the leakage current paths underneath the metal contact region [19–25]. With decreasing channel length, the leakage current gradually increases and ultimately leads to the device failure. Therefore, the fabrication of vertical devices with short-channel length using 2D semiconductors is still a serious challenge.

In this work, we report a simple and efficient metal transfer technique that used to fabricate high-performance vertical WSe_2_ devices. Predeposited metal electrodes are peeled off with PDMS/PVA stamp and then transferred on top of WSe_2_, so that a pristine channel and a clean metal–semiconductor interface can be obtained, which enabled the tunability of p-type and n-type behaviors of WSe_2_ transistors by using metals with different work functions. With this method, we fabricate vertical WSe_2_ FETs with a 18-nm-thick channel and a vertical WSe_2_ photodiode with graphene and Pt as asymmetric vdW contacts. The WSe_2_ photodiode exhibit a high rectification ratio (>10^5^) and excellent self-powered photodetection performance, demonstrating an photo-to-dark current ratio up to 1.5 × 10^5^, photoresponsivity of 0.28 A W^−1^, and detectivity of 1.35 × 10^10^ Jones, together with fast response speed of 24 μs, which are proved to be one of the best performance for vertical photodiodes. This work validates the importance of metal–semiconductor contact and provides a new solution for the construction of high-performance short-channel vertical photodiode.

## 2 Experimental section

### 2.1 The preparation, release, and transfer process of patterned metal electrodes

**Figure 1:**
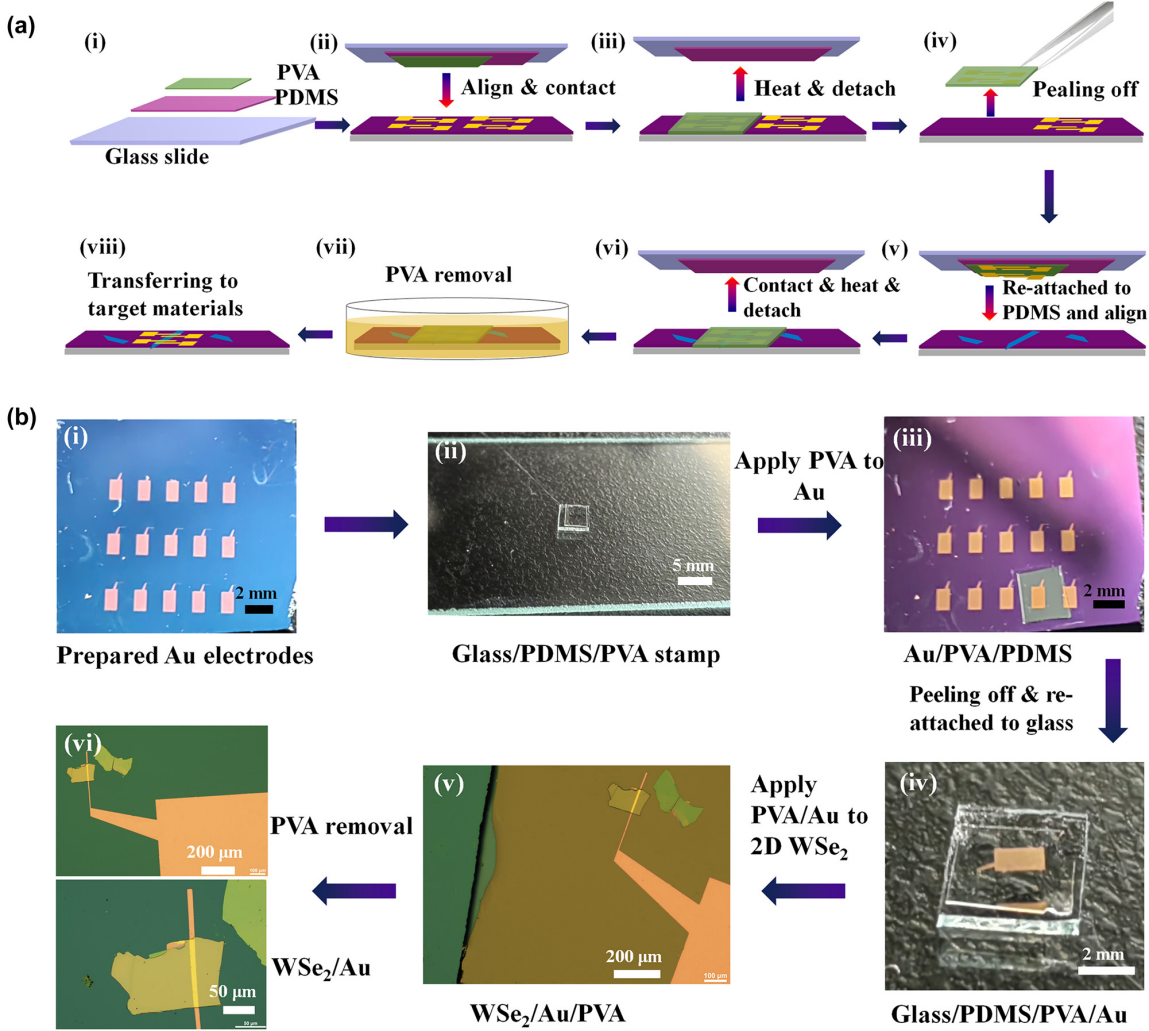
The fabrication processes of WSe_2_ transistors using PVA-assisted metal transfer technique. (a) Schematic illustration of the PVA-assisted metal transfer technique. (b) Photographs and optical micrograph (OM) images of metal electrode transfer process.

A series of 50-nm-thick metal electrodes with different work functions, including Pt (*ϕ*_m_ ∼ 5.5 eV), Au (*ϕ*_m_ ∼ 5.1 eV), In (*ϕ*_m_ ∼ 4.12 eV), and Al (*ϕ*_m_ ∼ 4.06 eV) (ZhongNuo Advanced Material [Beijing] Technology Co., Ltd), were firstly deposited on a sacrificial SiO_2_/Si chips following standard photolithography, thermal evaporation (sputtering for Pt), and lift-off procedures. The poly(vinyl alcohol) power (PVA, Sigma-Aldrich) with a molecular weight of 9000∼10,000 was dissolved into deionized water with a weight ratio of 10 %. The PVA aqueous solution was then dropped on a CD disk and baked at 70 °C for 1 h to form a thin film. Afterward, the PVA was cut into small pieces and brought into conformal contact with the PDMS stamp to form the transfer holder (Figure 1a(i)), which was then attached to a glass slide and clamped onto the arm of 3D transfer platform (Shanghai Onway technology Co., Ltd).

The release and transfer process for metal electrode is schematically illustrated in Figure 1a. A Si/SiO_2_ chip containing metal electrodes was placed onto the stage of the transfer platform, and the desired metal electrode was centered in the field of view. By monitoring the process with a microscope, the PDMS/PVA stamp can be precisely aligned and contacted with the underlying target metal electrodes, as illustrated in Figure 1a(ii). After that, the stage was heated to 90 °C for 2 min to fully melt the PVA film, which could not only ensure PVA adheres firmly to the underlying metal electrodes but also weaken the adhesion between the PDMS and PVA film. Once the PVA layer has fully melted, the arm of the transfer platform is lifted up slowly, making the PDMS stamp peeled off from the PVA, which adhered to the target substrate (Figure 1a(iii)). After cooling down to room temperature, the PVA film was solidified and the PVA film carrying the metal electrodes can be picked up from the substrate with a tweezer as shown in Figure 1a(iv). The stronger interaction between PVA/metal electrodes compared to that between metal electrode/substrate ensures the reliable peel-off of the metal electrode. The obtained PVA/metal can be reattached to PDMS stamp to form a new stack of glass slide/PDMS/PVA/metal electrode.

The glass slide/PDMS/PVA/metal electrode was mounted on the transfer platform and lowered slowly until it contact with the target WSe_2_ flake under an optical microscope (Figure 1a(v)). For the drop-down of metal electrode, the substrate is heated to 90 °C to make the PVA fully melted, giving rise to the strong contact between PVA and substrate together with the favored vdW adhesion of WSe_2_/metal electrode, and then the glass slide is raised slowly with the PVA carrying metal electrodes left on the substrate (Figure 1a(vi)). Finally, the PVA layer was removed by being dissolved in deionized water, and the metal electrodes were successfully transferred to the target WSe_2_ (Figure 1a(vii) and (viii)). The above pick-up and drop-off steps can also be used for constructing WSe_2_/graphene heterojunction.

### 2.2 The fabrication of planer and vertical WSe_2_ transistors

In order to compare the differences between conventional evaporated and transferred metal electrode, two planer WSe_2_ transistors with the same thickness were fabricated, one with the evaporated Au electrodes and another with the transferred Au contacts. To fabricate the vertical WSe_2_ photodiode, the Pt electrodes (50 nm) were firstly deposited on a SiO_2_/Si substrate by magnetron sputtering. Next, a multilayer WSe_2_ flake was transferred on top of Pt using a PDMS/PVA stamp. After that, a graphene flake and Au electrode was successively transferred onto the Pt/WSe_2_, resulting in vdW Pt/WSe2/graphene/Au heterostructure.

## 3 Results and discussion

The key to the successful transfer of metal electrode is the use of the bilayer film (PDMS/PVA) as a stamp. As reported in previous work, the PMMA [22, 26] and PPC [27] could also be used as the transfer mediator to transfer 2D materials; however, it usually cause polymer residuals and sample damage [28]. In addition, some volatile toxic solvents such as acetone and toluene are needed to remove the PMMA, which are harmful to human health. PVA is a small-molecule, water-soluble, and environment-friendly mediator, which can maintain the integrity and intrinsic properties of the samples after being removed. In addition, the PVA could enable selective transfer of the target sample, rather than the entire materials that prepared with CVD or mechanical exfoliation method.

The schematic illustration of the PVA-assisted metal transfer technique is shown in Figure 1a, and the corresponding photographs and OM images of the transfer process are shown in Figure 1b. With this method, contact electrodes can be laminated directly on the 2D materials without thermal damage. Therefore, a pristine channel and a clean vdW metal/semiconductor interface can be obtained. Due to PVA could be easily dissolved in hot water, the fabricated patterns of metal contacts can be well preserved, and there are no residues on the surface of the transferred metal electrodes and channel materials, as demonstrated in Figure 1b. Therefore, this method has the advantages of simplicity, efficient, and high accuracy. With this method, metal electrodes with different patterns can be easily transferred onto SiO_2_/Si substrates (Figure S1).

**Figure 2:**
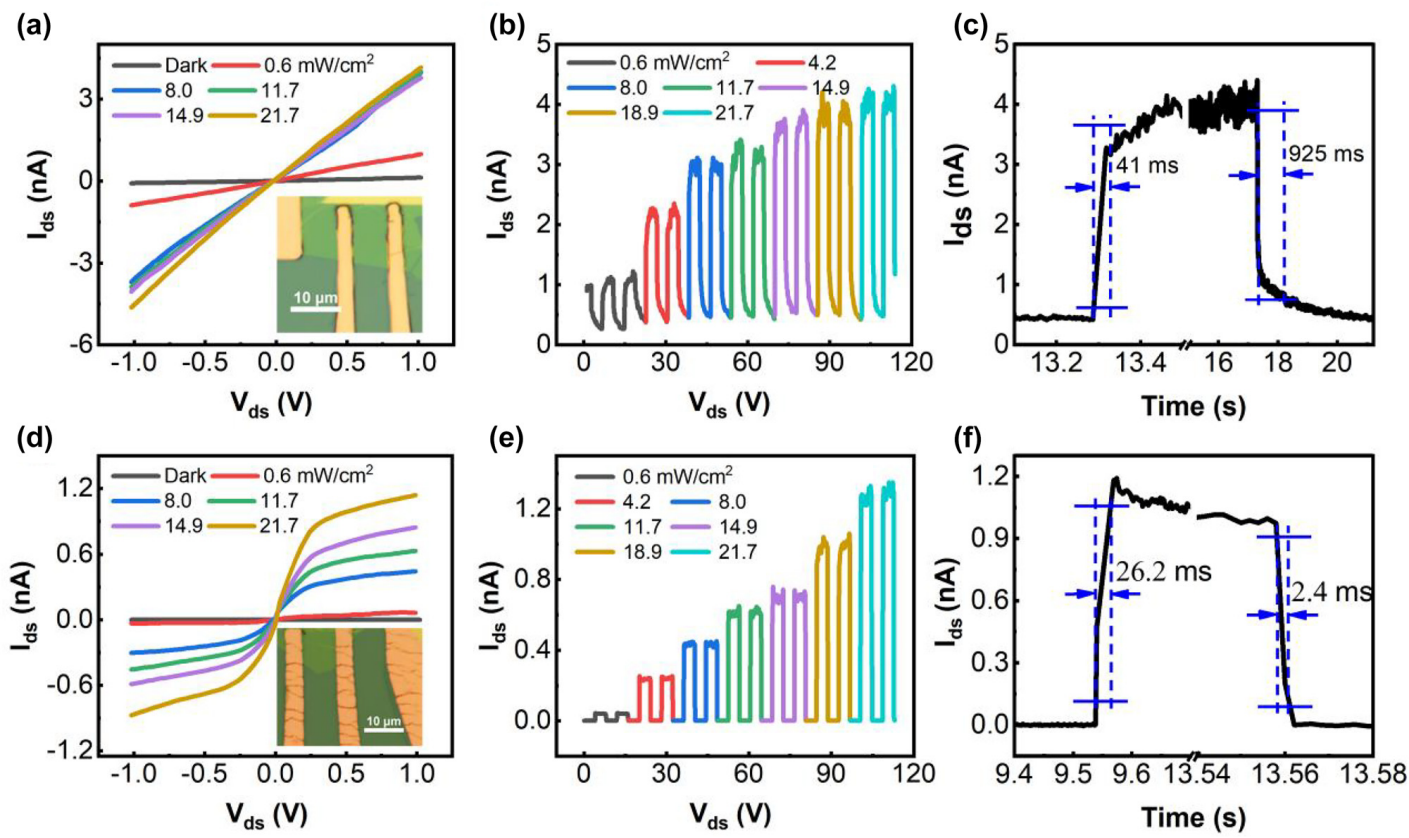
The *I*_d_–*V*_d_ curves of the WSe_2_ FETs with (a) evaporated and (d) transferred Au electrodes measured under dark and 405 nm laser irradiation with different intensities. (b) and (e) Are the corresponding time-resolved photoresponse of both devices at *V*_d_ = 1 V. (c) and (f) Are the rise and decay time of both devices acquired under pulsed illumination at *V*_d_ = 1 V.

In order to compare the optoelectronic performance between the devices with evaporated and transferred metal contacts, we fabricated two back-gated WSe_2_ field-effect transistors (FETs) with the similar thickness. One with the transferred Au electrodes as source and drain contacts, and another with thermally evaporated Au contacts. The OM images of both devices are shown in the inset of Figure 2a–d, and their thickness was estimated to be 5.1 and 4.3 nm, respectively, according to the AFM images shown in Figure S2. Figure 2d presents the *I*_d_–*V*_d_ curves of the WSe_2_ FETs with transferred Au electrodes under dark and 405-nm laser irradiation. The nonlinear *I*_d_–*V*_d_ curves indicate the existence of Schottky barrier at WSe_2_/Au interface, which is consistent with previous experimental results and theoretical simulation [19, 29], [30], [31]. By contrast, for device with evaporated Au contacts, the chemical interaction between Au atoms and WSe_2_ perturbs the electrical properties of WSe_2_. As explained in previous report [19], the surface of WSe_2_ was metalized under the Au electrode, which leads to an ohmic contact between Au and WSe_2_. Therefore, a linear *I*_d_–*V*_d_ curve was observed (Figure 2a). Compared with the device with transferred electrode, the one with evaporated Au has a higher photocurrent at the same laser intensity due to the lower contact resistance (Figure 2b–e). Furthermore, the photocurrent for the device with transferred Au electrode gradually increases with the increases of laser power owing to the enhancement of photoexcited carriers. While for the device with evaporated Au electrodes, the photocurrent saturates rapidly with increase of laser power owing to the trap filling.

Figure 2b–e shows the transient photoresponses of both devices under consecutive on/off light modulation under 405 nm laser illumination with *V*_d_ = 1 V. It is evident that the photocurrent of both devices increases with laser intensity, and they can be switched between on and off states with excellent reproducibility and stability. Benefiting from the ohmic contacts, the photodetector with evaporated Au contact has a higher photocurrent of about 4.2 nA at 21.7 mW cm^−2^ and *V*_d_ = 1 V. However, the relative large dark current and low *I*_light_/*I*_dark_ ratio (8.7) makes it unattractive for practical applications. Fortunately, the *I*_light_/*I*_dark_ ratio (642) was greatly improved by the introduction of Schottky contacts through transferring metal electrode, and the dark current was reduced from 475 pA for evaporated electrode to 1.8 pA for transferred contacts at *V*_d_ = 1 V. The response time *τ*, which is defined as the time for the photocurrent to rise/fall from 10/90 % to 90/10 % of the peak value, is affected by many factors, including the metal–semiconductor contact, trap, and defect states in the semiconductors. As shown in the transient photoresponse curve in Figure 2c and f, the photodetector with evaporated Au contact exhibits the rise/decay time of 41/925 ms. While for the vdW contacted device, the response time is significantly reduced to 26.2/2.4 ms. The reduced response time can be attributed to the Schottky contact that resulted in built-in fields at the Au/WSe_2_ interface, which are favorable for accelerating the separation and transport of photogenerated carriers, thus shortening the response times.

In order to investigate the influence of the work function of the contact metal, different WSe_2_ FETs were fabricated with Pt (*ϕ*_m_ ∼ 5.5 eV), Au (*ϕ*_m_ ∼ 5.1 eV), In (*ϕ*_m_ ∼ 4.1 eV), and Al (*ϕ*_m_ ∼ 4.05 eV) [18, 19, 31], [32], [33], [34] contacts using the same transfer method. Figure S3 shows the transfer curves of these back-gated WSe_2_ FETs. It was found that the charge transport properties of WSe_2_ FETs can be tailored by changing the work function of the transferred contact metals. It was changed from p-type (Pt) to p-type dominant ambipolar (Au) and finally to n-type dominant ambipolar (In and Al), as the work function of the metals decreasing. This result is accordance with previous reports and can be explained by the band diagram of metal–semiconductor [18, 19, 31], [32], [33], [34]. Pristine multilayer WSe_2_ is an ambipolar semiconductor with Fermi level (*E*_F_) close to the middle of the band gap, which can be tuned either toward conduction or valence band by contacting with different metals. According to the Schottky–Mott rule, n-type/p-type charge transport behavior would be dominant in WSe_2_ FETs contacting metals with low/high work function due to the low electron/hole Schottky barrier height. In comparison, previous reports have demonstrated that the WSe_2_ FETs with evaporated metals exhibited similar n-type unipolar characteristics regardless of work function owing to the strong Fermi-level pinning (FLP) [18, 32]. In a word, the PVA-assisted metal transfer method could overcome the FLP effect and achieve a clean and vdW WSe_2_–metal interface.

To demonstrate the significance of the metal/semiconductor contact properties to the performance of the vertical devices, vertical WSe_2_ FETs were fabricated with Au as metal contacts. The bottom Au electrode was prepared with conventional thermal evaporation technique, while the top Au contact was transferred on top of the WSe_2_ flake. For comparison, another Au electrode is also deposited on the same WSe_2_ flake using thermal evaporation method (Figure S4a and b). As shown in Figure S4d, the WSe_2_ FET contacted with evaporated Au electrodes displays a linear *I*_d_−*V*_d_ curve, with the dark current as high as 0.86 μA at *V*_d_ = 1 V, while the photocurrent under 405 nm illumination (25.7 mW cm^−2^) is too weak to be distinguished. Therefore, there is no photoresponse was observed under a series of periodical light stimulation via 405 nm laser (Figure S4f). For the vdW contacted vertical WSe_2_ device, the nonlinear *I*_d_–*V*_d_ curves confirm the existence of Schottky barrier at WSe_2_ and transferred Au interface, which leads to the dramatically reduced dark current of 1.53 nA under 1 V bias and enhanced photoresponse, as shown in Figure S4b. The photocurrent increases with laser intensity and the on/off ratio reaches 308 under 405 nm laser illumination (Figure S4e). This result confirms that the transferred contacts could minimize the damage of metal electrode to the 2D channel materials and reduce the dark current of vertical devices.

**Figure 3:**
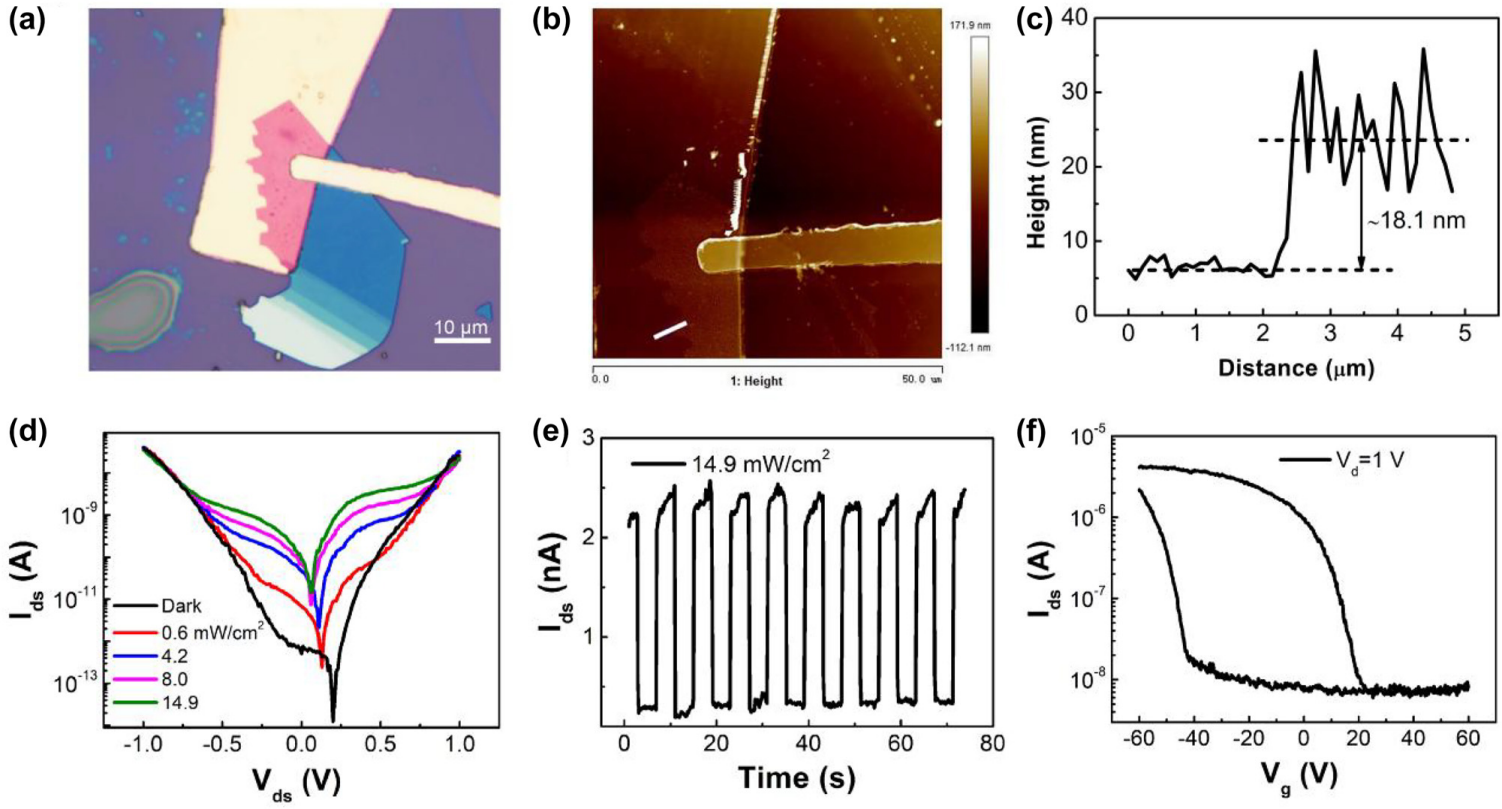
The (a) OM and (b) AFM image of the vertical short-channel WSe_2_ FETs. (c) The height profile along the white line in panel b indicates the thickness of WSe_2_ flake was ∼18.1 nm. (d) *I*_d_–*V*_d_ curves of the vertical WSe_2_ FETs under dark and 405 nm laser irradiation with different intensities. (e) The photoswitching characteristics of the WSe_2_ device under 405 nm laser irradiation at *V*_d_ = 0.5 V. (f) The transfer curve of the vertical WSe_2_ FETs under dark condition at *V*_d_ = 1 V.

Vertical short-channel WSe_2_ FETs was fabricated to demonstrate the potential of the PVA-assisted metal transfer method. The OM image of the device was shown in the inset of Figure 3a, and its thickness measured with AFM is 18.1 nm (Figure 3b and c). The *I*–*V* curves in logarithmic scale under dark condition and 405 nm laser irradiation are shown in Figure 3d. When the WSe_2_ device is biased with a low voltage (*V*_d_ < 0.65 V), the dark current remains at a low level (<1 nA) due to the back-to-back Schottky barrier. Hence, the illumination of 405 nm laser generate an obvious photocurrent, thereby achieving an on/off current ratio of nearly 10 under a light intensity of 14.9 mW cm^−2^ at *V*_d_ = 0.5 V, as shown in Figure 3e. When the reverse bias voltage reaches a certain value (0.7 V in Figure 3d), the dark current increases rapidly and almost coincides with photocurrent, indicating the breakdown of the Schottky junction.

To evaluate the gate modulation ability of the vertical WSe_2_ device, the transfer characteristic was measured. Figure 3f shows the double-sweep transfer curve of the vertical short-channel WSe_2_ device at *V*_d_ = 1 V. Although bottom Au electrode may partially shields the vertical electric field effect to the upper WSe_2_ channel, the transfer curve measurement revealed that the device still retain gate coupling effect with on/off ratio 5.6 × 10^2^, which may be caused by the large area of the WSe_2_ flakes that some part of the WSe_2_ is in direct contact with the SiO_2_/Si substrate. In addition, the vertical WSe_2_ transistor has a hysteresis window of 60 V under a gate voltage of ±60 V, which indicates the potential of the device for memory applications, and previous studies have demonstrated that the water and oxygen molecules trapped between SiO_2_/Si substrate and WSe_2_ channel plays the dominant role in the hysteresis of the transfer curves [35, 36].

**Figure 4:**
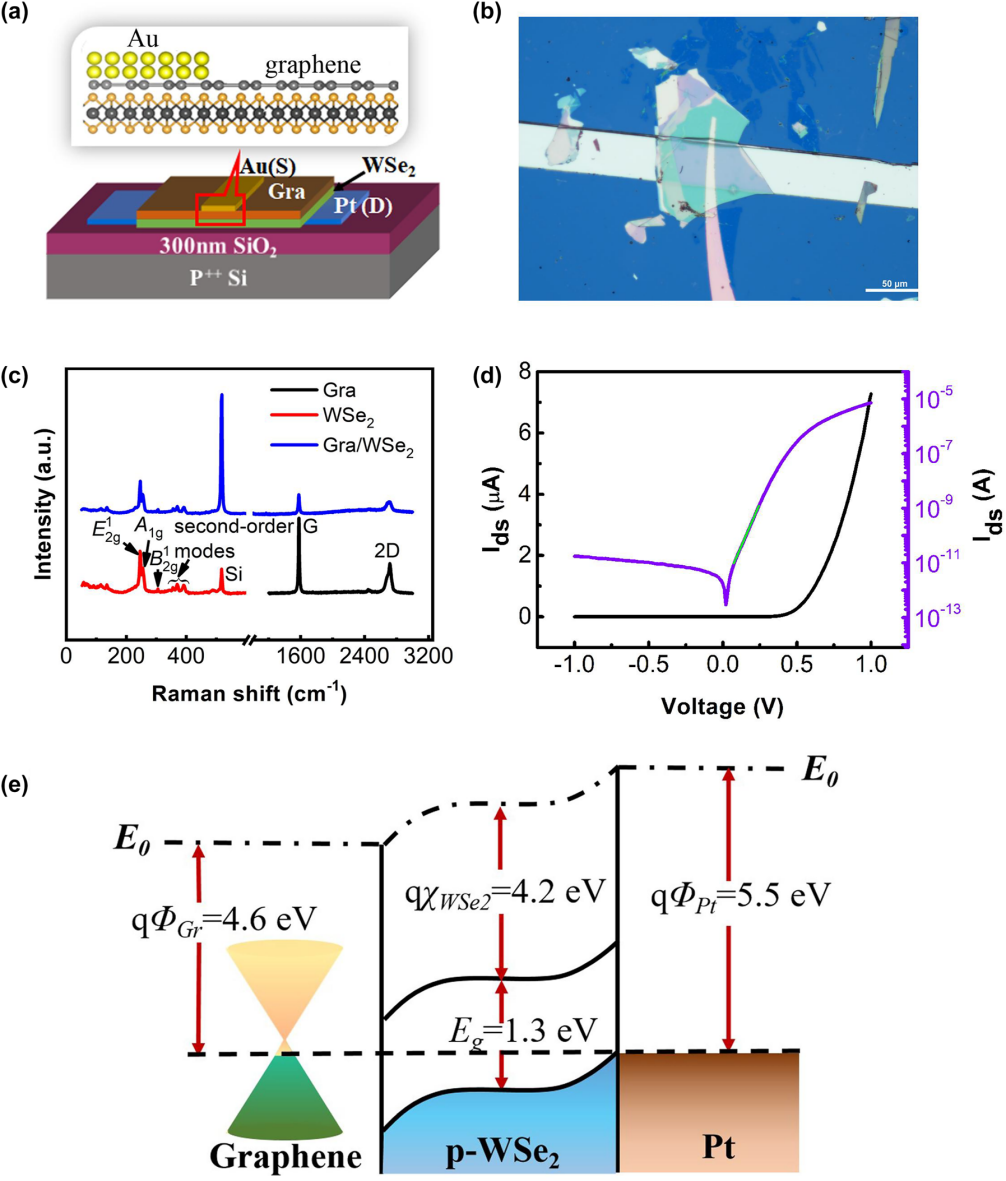
Device structure and characterization of vertical WSe_2_ photodiode. (a) Schematic and (b) OM image of the vertical Pt/WSe_2_/graphene/Au FETs. (c) Raman spectra of individual WSe_2_, graphene, and the WSe_2_/graphene heterojunction with 532 nm laser excitation. (d) *I*_d_–*V*_d_ curve of the vertical Pt/WSe_2_/graphene/Au in logarithmic and liner scale under dark condition. (e) Energy band diagram of the vertically stacked Pt/WSe_2_/graphene heterostructures.

In order to further improve the photoresponse performance of the vertical WSe_2_ device, we fabricated a vertical Pt/WSe_2_/graphene/Au photodiode. As shown in Figure 4a, the device is constructed by vertically sandwiching multilayer WSe_2_ between the top graphene and the bottom Pt electrode. Figure 4b displays the OM of the vertical heterostructure device. The effective area of WSe_2_ between the graphene and Pt is calculated to be about 3159 μm^2^. The Raman spectrum of the vertical WSe_2_/graphene heterojunction exhibits five main peaks (Figure 4c). The peaks located at 245.87, 254.89, and 305.25 cm^−1^ correspond to the E_2g_^1^, A_1g_, and B_2g_^1^ modes of multilayer WSe_2_, respectively [12]. While the peaks appeared at 1579.26 and 2714.81 cm^−1^ can be ascribed to the G and 2D phonon modes of graphene, respectively [37]. There is no shift for the characteristic Raman peaks of both WSe_2_ and graphene in the heterojunction compared to the spectrum of individual crystal, suggesting good quality of each component after transfer and device fabrication process. The AFM image of the heterojunction device is shown in Figure S5, and the thickness of WSe_2_ and graphene are 58.9 and 16.7 nm, respectively.

Figure 4d shows the *I*_d_–*V*_d_ curve of the vertical device at a bias voltage range of *V*_d_ = ±1 V measured in dark. The bottom Pt electrode is defined as drain electrode while the Au on graphene as source electrode. The vertical device exhibits strong rectification behavior with a large forward current over 7.1 μA at *V*_d_ = 1 V and a low reverse current below 18.4 pA at *V*_d_ = −1 V, and thus a very high current rectification ratio of 3.9 × 10^5^ at |*V*_d_| = 1 V was obtained, which is superior to most vdWs 2D heterostructures reported in recent literatures [11, 12, 38, 39]. The *I*–*V* curve can be fitted with the Shockley diode equation: *I* = *I*_s_(*e*^*qV*/*nKT*^−1), where *I*_s_ is the reverse saturation current, *n* is the ideality factor, *q* is the elementary charge, *K* is the Boltzmann constant, and *T* is the absolute temperature. The ideality factor that determined from the slope of linear region of the *I*–*V* curve in Figure 4d is 1.52. This value is slightly higher than ideal value (*n* = 1), but smaller than many reported Schottky photodetectors, indicating good junction interface. The energy band diagram of the Pt/WSe_2_/graphene heterojunction is illustrated in Figure 4e. According to previous reports, the bandgap and electron affinity of multilayer WSe_2_ are approximately 1.3 and 4.2 eV [12, 34, 40], respectively, and the work function of graphene and Pt are 4.6 and 5.5 eV, respectively [34, 40]. The work function of the bottom Pt electrode matches well with the valence band edge of WSe_2_, which provides an ohmic contact to WSe_2_ (Figure S6). Graphene, on the other hand, forms a vdW Schottky barrier after contacting WSe_2_, as demonstrated by previous experimental results and theoretical simulation [8, 40], [41], [42]. The difference in work function of Pt and graphene leads to a built-in potential in the device, which generates an excellent rectification ratio.

**Figure 5:**
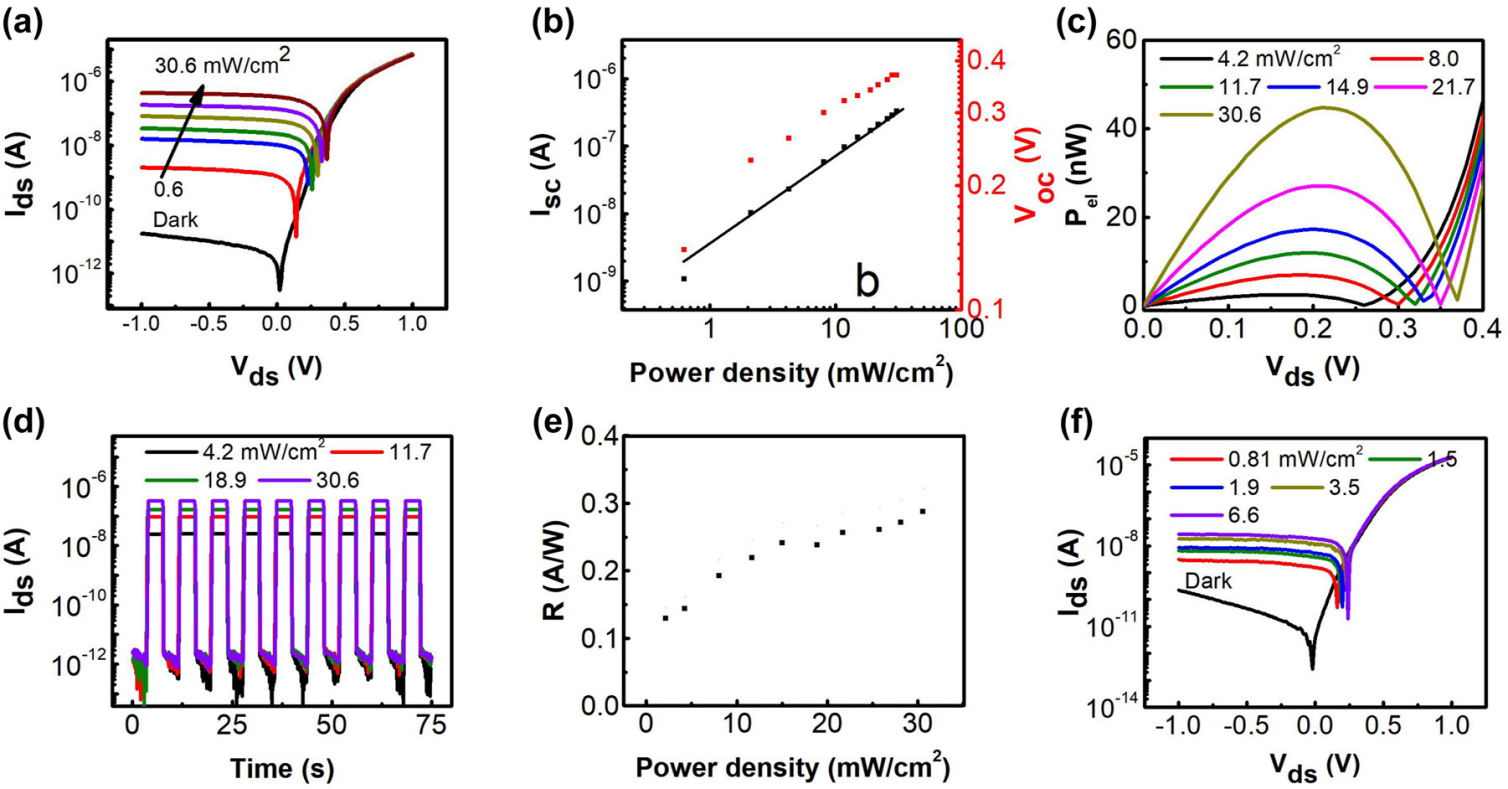
The optoelectronic properties of vertical WSe_2_ photodiode. (a) *I*_d_–*V*_d_ curves of the vertical Pt/WSe_2_/graphene/Au device under dark and 405 nm laser illumination with different intensities. (b) Power density–dependent short-circuit current (*I*_SC_) and open-circuit voltage (*V*_OC_). (c) The *V*_d_-dependent output electrical power (*P*_el_) under 405 nm light illumination. (d) The photoswitching characteristics of the device at zero bias under 405 nm laser illumination. (e) Power-dependent responsivity of the device at zero bias. (f) The *I*_d_–*V*_d_ curves of the vertical Pt/WSe_2_/graphene/Au device under dark and 808 nm laser illumination with different intensities.

Subsequently, the optoelectronic properties of the vertical Pt/WSe_2_/graphene/Au photodiode were investigated to examine its potential as a photodetector. The *I*_d_–*V*_d_ curves on a logarithmic scale under dark and 405 nm laser illumination were shown in Figure 5a, with the corresponding linear-scale curves plotted in Figure S7. Apparently, the *I*_d_–*V*_d_ curves are upshifted under laser illumination, exhibiting a distinct photovoltaic response, which demonstrates the device has a self-powered detection feature. When the device is illuminated with 405 nm laser, electron–hole pairs are excited in both graphene and WSe_2_. The *V*_OC_ and *I*_SC_ were extracted and plotted as a function of the power density in Figure 5b, and the maximum *V*_OC_ of 0.37 V and *J*_SC_ of 331 nA were generated under a light intensity of 30.6 mW cm^−2^. Therefore, the vertical Pt/WSe_2_/graphene/Au device shows remarkable photovoltaic behavior under the 405 nm illumination. In addition, the *I*_SC_ followed a logarithmic dependence on the light intensity, which can be fitted by the equation: *I*_SC_ ∝*P*^*θ*^, where *θ* is a power law exponent that related to the recombination processes of the photogenerated carriers [43], and *P* is the light intensity. By fitting the experimental data, *θ* is deduced to be 1.28. For the ideal case, the value of *θ* is equal to 1, which implies all the photoexcited electron–hole pairs contributed to the photocurrent without any trapping and recombination [44]. Practically, the value of *θ* is usually lower than 1, and *I*_ph_ usually exhibits a sublinear dependence on light intensity due to some unwanted recombination. However, in this work, an unusual superlinear light intensity dependence of photocurrent was observed (*θ* > 1), and it features increased photoresponsivity with power density. Similar superlinear behavior has been recently observed in a series of 2D semiconductors and vdW heterojunctions, such as graphene [45], Ta_2_NiSe_5_ [46], graphene/h-BN [47], and WS_2_/MoS_2_ heterojunctions [48]. The typical origin of superlinearity can be attributed to the photothermionic (PTI) emission. The photon energy absorbed by graphene could generate a thermalized hot carrier distribution, and those carriers with an energy larger than the Schottky barrier at the WSe_2_/graphene interface can be injected into WSe_2_ [49], which could significantly extend the light response range and enhance the photoresponsivity.

The output electrical power *P*_el_, defined as *P*_el_ = *I*_d_ × *V*_d_, was plotted in Figure 5c. The maximum *P*_el_ could reach 44.7 nW at a bias voltage of 0.21 V when light intensity is 30.6 mW cm^−2^, corresponding to a power conversion efficiency of 4.6 %, which is comparable to or higher than that of most vdW heterojunction solar cells [8, 16, 17]. To investigate the photoswitching behavior of the vertical Pt/WSe_2_/graphene/Au device under light illumination, Figure 5d exhibits the multicycle *I*–*t* curves of the device under 405 nm laser illuminations with various intensities at zero bias. It is evident that the device could be switched between on and off states with excellent reproducibility and stability. In particular, the drain current increases rapidly from about 2.3 pA at dark condition to 0.33 μA, thereby achieving an ultrahigh on/off current ratio of ∼1.4 × 10^5^ under a light intensity of 30.6 mW cm^−2^, which signifies outstanding photoresponse of the device to the visible light.

To further evaluate the optoelectronic performance of the vertical Pt/WSe_2_/graphene/Au device as a photodetector, the responsivity (*R*) is calculated by the following equation:

$$R=\left(I_{\text{light}}-I_{\text{dark}}\right)/P\cdot S$$

where *I*_light_, *I*_dark_, *P*, and *S* are the photocurrent, dark current, incident power intensity, and effective area of the device, respectively.

Based on the above formulas, the calculated *R* of the Pt/WSe_2_/graphene/Au device under various power densities at zero bias is shown in Figure 5e. A decent *R* of 0.28 A W^−1^ was achieved at a power density of 30.6 mW cm^−1^. Simultaneously, the responsivity increases slowly with increasing incident power, which further confirms the superlinear behavior. In addition to responsivity, the specific detectivity (D*) is another significant parameter that reflects the ability of a photodetector to detect weak signal, which can be calculated by the equation:

$$D*=\frac{\sqrt{S_{\text{device}}}}{\text{NEP}}=\sqrt{\frac{S_{\text{device}}}{S_{\text{id}}}}\times R$$

where NEP is the noise equivalent power, *S*_device_ is the device area, and *S*_id_ is the noise power spectral density of the detector. To accurately characterize D*, the noise power spectral density is measured in dark condition and shown in Figure S8, which displays a typical 1/*f* power density in the low frequency range. At small measurement bandwidth of Δ*f* = 1, the D* is calculated to be 1.35 × 10^10^ Jones.

Next, the photoresponse performance of this device upon excitation by a 532 and 808 nm laser was investigated. Figures S9 and 5f exhibit the corresponding dark and photo-induced *I*–*V* curves of the WSe_2_ photodiode on a logarithmic scale. Because WSe_2_ multilayers have a bandgap of 1.3 eV, the WSe_2_ photodiode can respond to NIR light even though the response is weaker than that to visible light. The device displays an obvious photovoltaic behavior with a maximum *V*_OC_ of 0.25(0.3) V and *I*_SC_ of 18(71) nA to 808(532) nm laser. The time-resolved photoresponse at zero bias under different light intensities is shown in Figures S10 and S11. The device shows excellent photocurrent switching behaviors and negligible changes under periodic 532 and 808 nm laser illuminations, and the current on/off ratio decreases from 2.7 × 10^3^ for 532 nm laser to 4.0 × 10^2^ for 808 nm laser, demonstrating the device has a self-powered photodetection feature from visible to NIR light.

**Figure 6:**
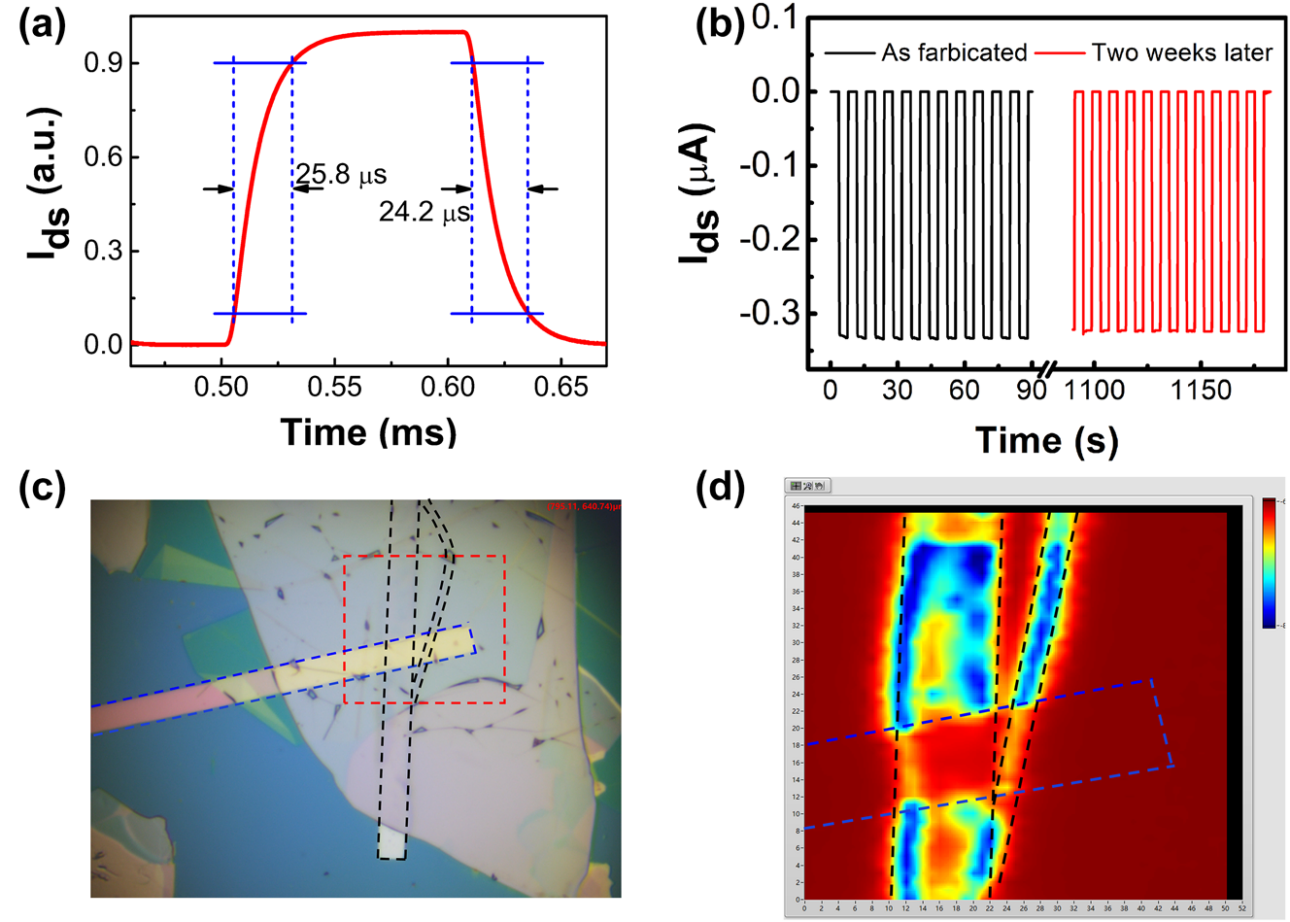
The temporal response, stability, and photocurrent mapping of vertical WSe_2_ photodiode. (a) Rise and decay time of the Pt/WSe_2_/graphene/Au device measured at *V*_d_ = 0 V under the illumination of 520 nm laser. (b) The photoresponse of the device was monitored before and after stored in an airtight container for 2 weeks. (c) The OM image of the vertical Pt/WSe_2_/graphene/Au device. The top Au and bottom Pt electrode are outlined by blue and black dotted lines, respectively. (d) Scanning photocurrent mapping at zero bias with an illumination wavelength of 520 nm.

The photoresponse speed of the device to 532 nm laser at zero bias voltage was recorded with an oscilloscope. The rise/decay time defined as the time taken for photocurrent increasing/decreasing from 10/90 % to 90/10 % of the maximum value was calculated to be 25.8/24.2 µs (Figure 6a), indicating the fast separation of the photogenerated electron–hole pairs under the internal electric field. For practical applications, the long-term working stability is another key parameter for a high-performance photodetector. The vertical Pt/WSe_2_/graphene/Au device exhibits excellent long-term stability by storing it in an airtight container. The photocurrent exhibits no noticeable deviation and the on/off ratio decrease slightly after about 2 weeks (Figure 6b). To study the photosensitive region of the device, scanning photocurrent measurement was carried out with a confocal optical microscope. The focused 532 nm laser spot was scanned over the sample, and the resultant photocurrent at zero bias was recorded as a function of laser spot position. The photocurrent map presented in Figure 6d indicates that the photocurrent is mainly generated in the region where the three main layers (Pt/WSe_2_/graphene) are superimposed, which could be attributed to the efficient separation and transportation of photogenerated electron–hole pairs by built-in electric field. There is no current generated in the area covered by the Au electrode as it blocks the entrance of incident light into the WSe_2_ layer. No distinct photocurrent generation away from the Pt/WSe_2_/graphene overlapping region, which may be due to the photocarriers generated in the WSe_2_/graphene far away from the Pt electrode cannot be effectively collected.

## 4 Conclusions

In summary, we reported an effective PVA-assisted metal transfer technique that enables predeposited metal electrode to be reliably peeled off and transferred onto target 2D materials. Compared to traditional evaporated contacts, this transfer printing method could reduce Fermi-level pinning and enables the tunability of p-type and n-type WSe_2_ transistors by using metals with different work functions. With this technique, a vertical short-channel WSe_2_ FETs and a vertical WSe_2_ photodiode with asymmetric graphene and Pt vdW contacts were fabricated. The WSe_2_ photodiode exhibited superior current rectification exceeding 10^5^ and excellent self-powered photoresponse characteristics, including an photo-to-dark current ratio up to 1.5 × 10^5^, photoresponsivity of 0.28 A W^−1^, and detectivity of 1.35 × 10^10^ Jones, together with fast response speed of 24 μs, which are among the highest values ever reported for vertical photodiodes. This work validates the importance of metal–semiconductor contact and provides a new solution for the construction of high-performance short-channel vertical photodiode.

## Supporting Information

The Au electrodes with different patterns that before and after transfer with PDMS/PVA stamp; AFM analysis of the WSe_2_ FETs with evaporated and transferred Au electrodes; the transfer curves of WSe_2_ FETs with different metal contacts; the optoelectronic properties of the vertical WSe_2_ FETs prepared with evaporated and transferred metal methods on the same WSe_2_ flakes; AFM analysis of the Pt/WSe_2_/graphene/Au FETs; *I*–*V* curves of the two terminal Pt–WSe_2_–Pt device under dark and 405 nm laser illumination; *I*_d_–*V*_d_ curves of the vertical Pt/WSe_2_/graphene/Au device under dark and 405 nm laser illumination; *I*_d_–*V*_d_ curves of the vertical Pt/WSe_2_/graphene/Au device under dark and 532 nm laser illumination; the photoswitching characteristics of the device at zero bias under 532 and 808 nm laser irradiation.

## Supplementary Material

Supplementary Material Details
