# Supplementary material for: PVA-assisted metal transfer for vertical WSe2 photodiode with asymmetric van der Waals contacts
Source: Nanophotonics. 2023 Aug 28;12(18):3671–82. doi: 10.1515/nanoph-2023-0398 (PMC11502030; doi:10.1515/nanoph-2023-0398)
Supplement: Supplementary file 1 — Supplementary Material Details [file j_nanoph-2023-0398_suppl_001.docx]

# PVA-assisted metal transfer for vertical WSe_2_ photodiode with asymmetric van der Waals contacts

Xiaohui Song,^1^ Zhen Liu^1^, Zinan Ma,^1^ Yanjie Hu,^1^ Xiaojing Lv,^1^ Xueping Li,^2^ Yong Yan,^1^ Yurong Jiang,^1^ Congxin Xia^1,^^[[1]](#footnote-0)^*

^1^Henan Key Laboratory of Photovoltaic Materials, School of Physics, Henan Normal University, Xinxiang 453007, China

^2^Department of Electronic and Electrical Engineering, Henan Normal University, Xinxiang 453007, China


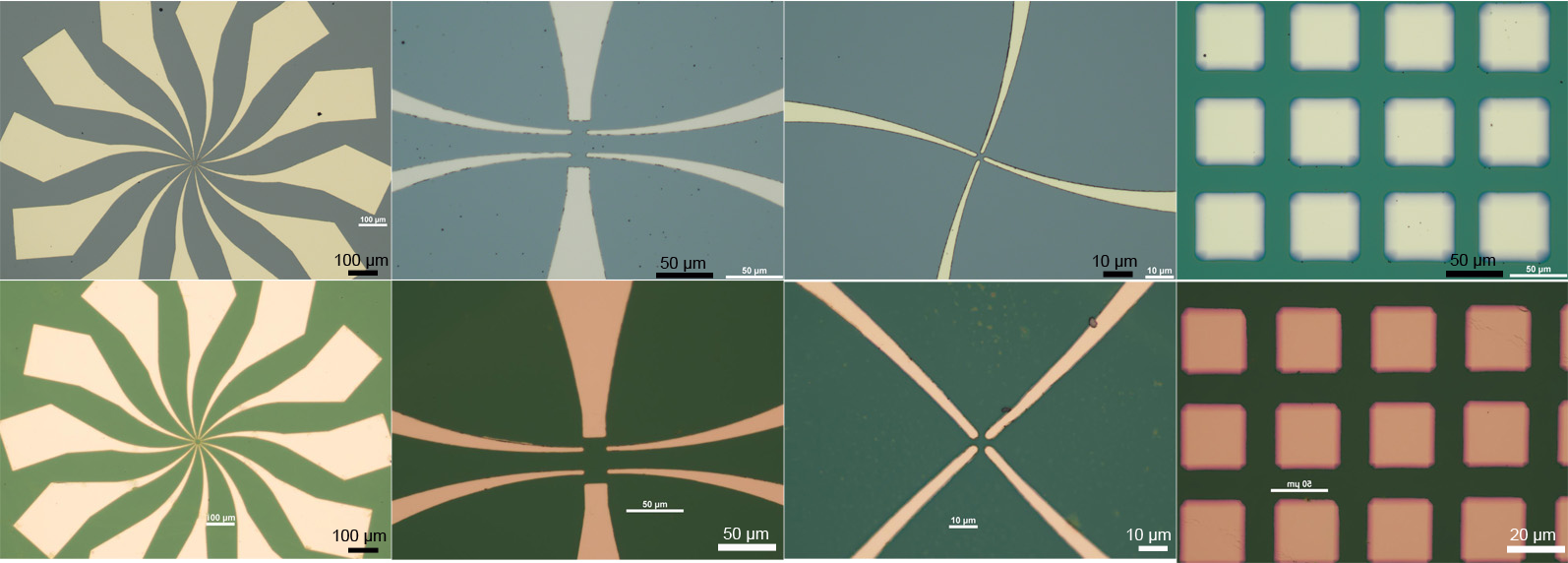


**Figure S1**. The Au electrodes with different patterns that before (a-d) and after (e-h) transfer with PDMS/PVA stamp.


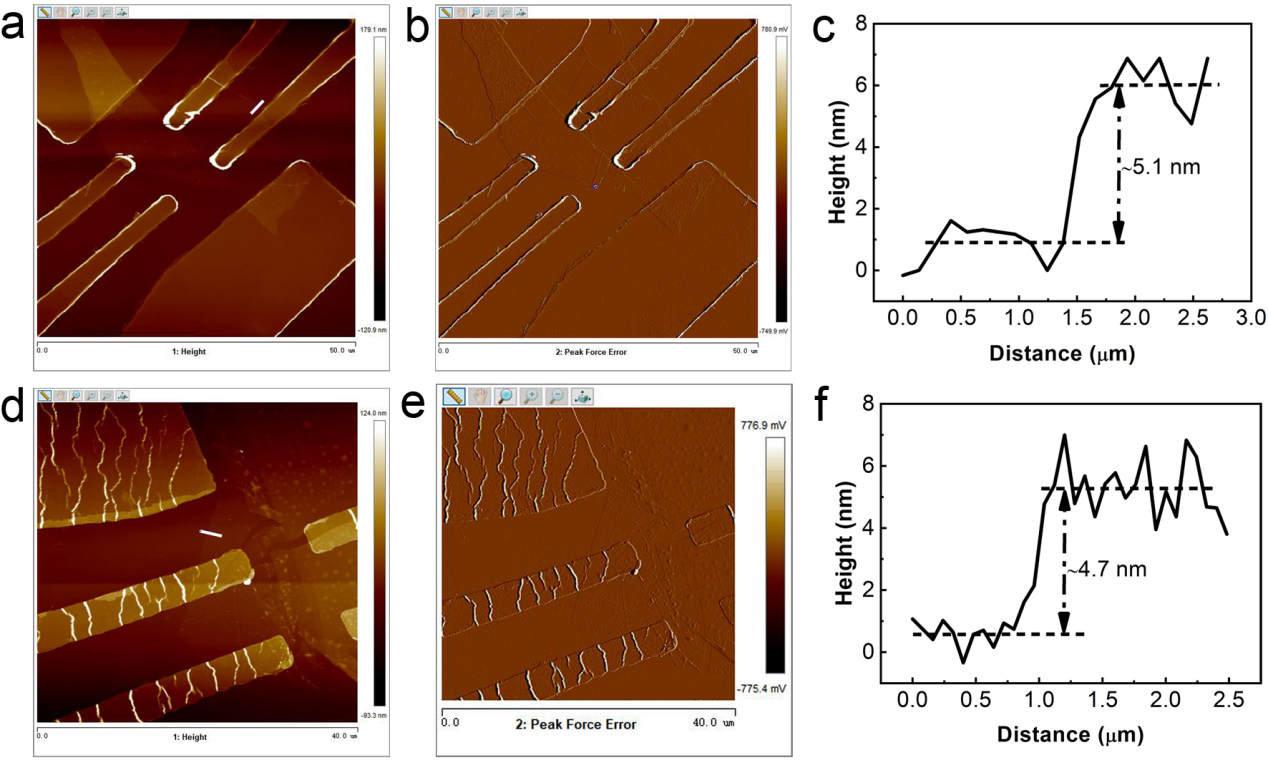


**Figure S2.** AFM image of the WSe_2_ FETs with (a) evaporated and (d) transferred Au electrodes. Panel (b) and (e) are the corresponding 2D AFM images of both devices. The thickness of the WSe_2_ FETs with (c) evaporated and (f) transferred Au electrodes, as scanned along the white line in panel (a) and (d), is about 5.1 and 4.7 nm, respectively.





**Figure S3**. The transfer curves (I_d_-V_g_) of WSe_2_ FETs with (a) Pt, (c) Au, (e) Al and (g) In contacts. The corresponding I_d_-V_g_ curves in logarithmic scale are shown in panel b, d, f, and h, respectively.


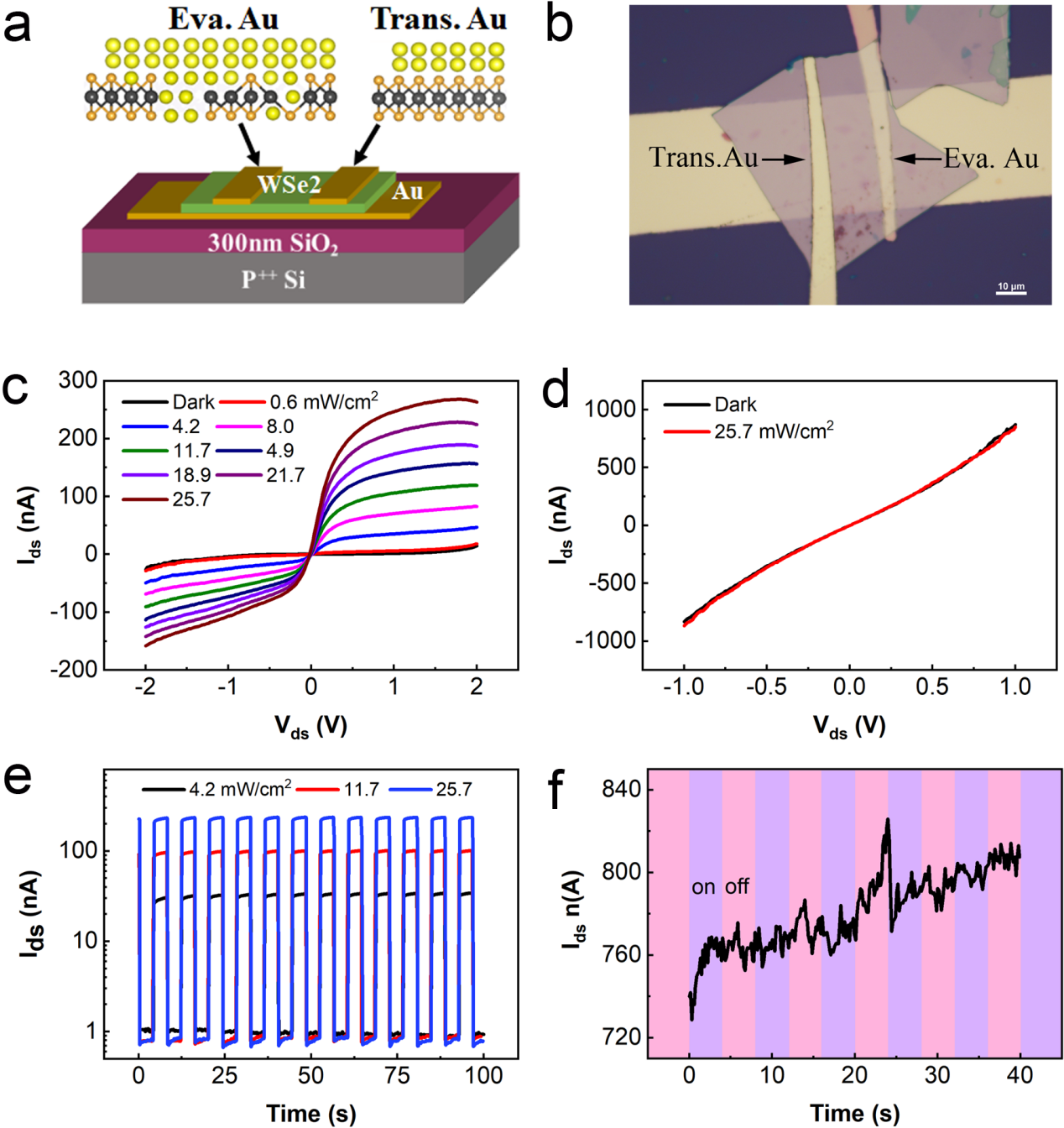


**Figure S4.** (a) Schematic and (b) OM image of the vertical WSe_2_ FETs prepared with both conventional evaporated and transferred metal methods on the same WSe_2_ flakes. I_d_-V_d_ curves of the vertical WSe_2_ FETs with (c) transferred and (d) evaporated Au electrode measured under dark and 405 nm laser irradiation with different intensities. Time-resolved photoresponse of the WSe_2_ FETs with (e) transferred and (f) evaporated Au electrodes under different laser powers at V_d_=1 V.


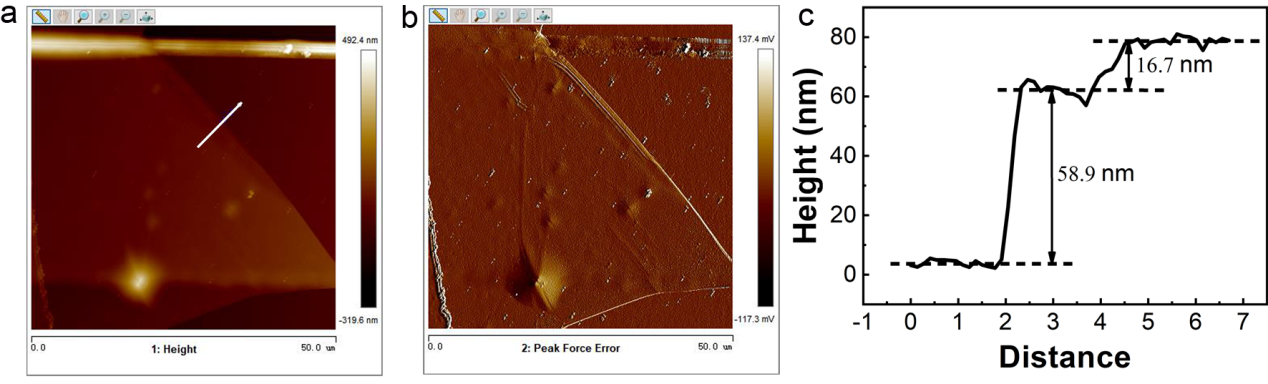


**Figure S5.** AFM image of the Pt/WSe_2_/graphene/Au FETs. Panel b is the corresponding 2D AFM images of the device. The thickness of the WSe_2_ and graphene flake, as scanned along the white line in panel a, is about 58.9 and 16.7 nm, respectively.


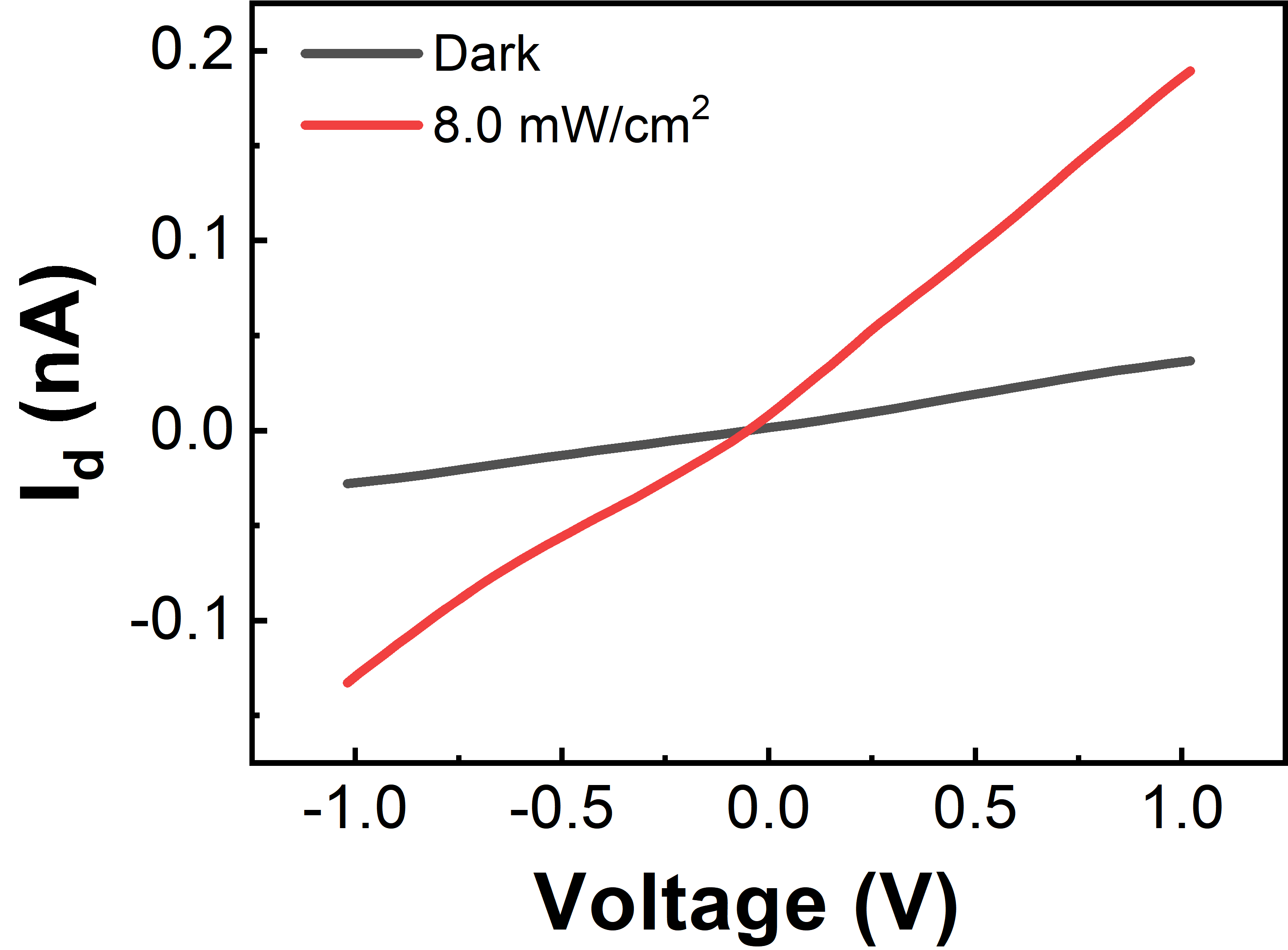


**Figure S6.** I-V curves of the two terminal Pt-WSe_2_-Pt device under dark and 405 nm laser illumination.





**Figure S7.** I_d_-V_d_ curves of the vertical Pt/WSe_2_/graphene/Au device under dark and 405 nm laser illumination with different intensities.





**Figure S8**. The noise power spectral density of the fabricated Pt/WSe_2_/graphene/Au device measure at V_d_ = 0 V in dark condition.





**Figure S9.** I_d_-V_d_ curves of the vertical Pt/WSe_2_/graphene/Au device under dark and 532 nm laser illumination with different intensities.





**Figure S10.** The photoswitching characteristics of the device at zero bias under 532 nm laser irradiation with different power densities.





**Figure S11.** The photoswitching characteristics of the device at zero bias under 808nm laser irradiation with different power densities.

1. *E-mail address*: [xiacongxin@htu.edu.cn](mailto:xiacongxin@htu.edu.cn) (C. Xia) [↑](#footnote-ref-0)
